# Meta-analysis of XRCC1 polymorphism and risk of female reproductive system cancer

**DOI:** 10.18632/oncotarget.16090

**Published:** 2017-03-10

**Authors:** Na-Na Yang, Ying-Fan Huang, Jian Sun, Ying Chen, Zhong-Min Tang, Jin-Fang Jiang

**Affiliations:** ^1^ Nursing Department, Affiliated Tumor Hospital of Guangxi Medical University, Nanning, China; ^2^ Department of Medical Affairs, ZiBo Hospital of Integrated Traditional Chinese and Western Medicine, Zibo, China; ^3^ Radiology Department, Affiliated Tumor Hospital of Guangxi Medical University, Nanning, China; ^4^ Chemotherapy Department, Affiliated Tumor Hospital of Guangxi Medical University, Nanning, China

**Keywords:** XRCC1, gene polymorphism, female reproductive system cancer, meta-analysis

## Abstract

Numerous epidemiological studies have evaluated the association between polymorphism in the gene encoding x-ray repair cross complementing 1 (XRCC1) protein and the risk of female reproductive system cancer, but results are inconclusive. To gain a comprehensive picture of available evidence, we searched for relevant studies in the PubMed, EMBASE, Scopus, and Chinese National Knowledge Infrastructure databases up to December 17, 2016. A total of 26 case-control studies were picked out. The pooled odds ratio (OR) with its 95% confidence interval (CI) was calculated to estimate the association. Based on data of all study participants, we did not find a positive association of rs25487 or rs1799782 polymorphism with risk of female reproductive cancer risk. Subgroup analysis, however, identified two alleles as being associated with an increased risk of female reproductive system cancer in Asians: the A allele of rs25487 (heterozygous genetic model, OR 1.16, 95%CI 1.00–1.36), and the T allele of rs1799782 (homozygous model, OR 2.30, 95%CI 1.39–3.82; dominant model, OR 1.28, 95%CI 1.10–1.50; recessive model, OR 2.11, 95%CI 1.33–3.34). Moreover, the AA genotype at rs25489 was determined to be a risk factor for cervical cancer etiology (homozygous model, OR 2.91, 95%CI, 1.17–7.26; recessive model, OR 3.16, 95%CI 1.91–5.24). This meta-analysis suggests that no association between rs25487 or rs1799782 gene polymorphism and risk of female reproductive cancer risk was found. These results should be validated in larger studies.

## INTRODUCTION

Female reproductive system cancer, which includes cervical cancer, endometrial cancer, and ovarian cancer, is a major threat to women's health. In fact, cervical cancer ranks third among all gynecologic cancers in the world [1], 65 new endometrial cancer cases occur annually per 100,000 women between the ages of 65 to 75 [2], and approximately 140,200 new ovarian cancer cases worldwide per year are recorded [3]. Elucidating the etiology of female reproductive system cancer and identifying at-risk populations may allow more effective early detection and perhaps even prevention.

The causes of these cancers remain poorly understood. Infection with oncogenic human papilloma virus (HPV) is a risk factor in tumorigenesis [4], but many HPV carriers do not develop cervical cancer, indicating that there must be other cancer risk factors, such as genetic and environmental factors. One possible genetic factor may be polymorphism in the gene encoding x-ray repair cross complementing 1 (XRCC1) protein. The gene is located on chromosome 19 (19q13.2), and the expressed protein is involved in the base excision repair (BER) pathway [5, 6], which helps correct errors during DNA replication and recombination as well as preserve genome integrity [7]. Functional single-nucleotide polymorphisms (SNPs) in *XRCC1* have been linked to development of esophageal squamous cell carcinoma [8], lung cancer [9], pancreatic cancer [10], breast cancer [11], colorectal cancer [12], and gastric cancer [13]. While more than 300 *XRCC1* SNPs have been described in the dbSNP database, three functional SNPs have been extensively studied, all of which cause amino acid substitutions in the encoded protein: rs25487 [Arg399Gln], rs1799782 [Arg194Trp] and rs25489 [Arg280His].

Associations between *XRCC1* SNPs and risk of female reproducitve system cancer are unclear, because the several molecular epidemiologic studies conducted so far have been inconclusive. This lack of clarity may reflect the relatively small statistical power in individual studies, as well as heterogeneity in genetic backgrounds of study participants. Therefore we performed this meta-analysis to comprehensively assess available evidence on the association between *XRCC1* polymorphism and risk of female reproductive system cancer.

## RESULTS

### Study characteristics

**Figure 1:**
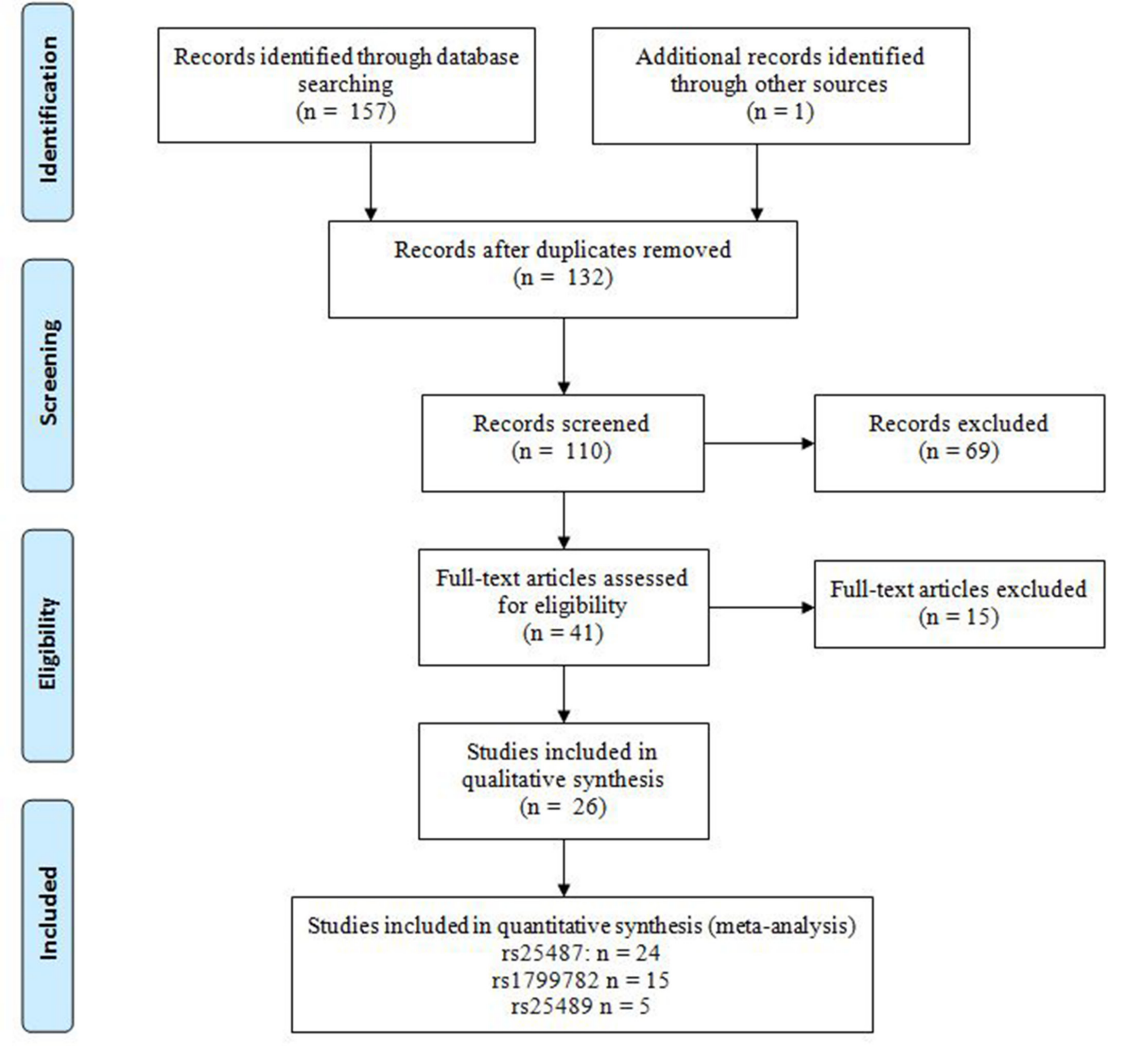
PRISMA flow diagram

Systematic search of the PubMed, EMBASE and China National Knowledge Infrastructure (CNKI) databases identified 157 potentially relevant studies (Figure 1). Further screening allowed elimination of all but 31 studies, which were read in full. In the end, 26 case-control studies were included in this study (Table 1): data on rs25487 were reported in 24 studies involving 4,265 cases and 5,495 controls; data on rs1799782 were reported in 15 studies involving 2,672 cases and 3,578 controls; and data on rs25489 were reported in 5 studies with 907 cases and 1,416 controls. The various ethnic groups involved in the studies were divided into two categories: Asian or Non-Asian, with the latter including Caucasian, Latino, and mixed.

**Table 1:** Characteristics of studies included in the meta-analysis

First author	Year	Country	Ethnicity	Cancer type	Sourcecon	Source of DNA	Genotyping method	Case	Control	HWEcon
***rs25487 (Arg399Gln)***										
Sobczuk	2012	Poland	Caucasian	Endometrial	HB	Blood	PCR-RFLP	94	114	0.161
Hosono	2013	Japan	Asian	Endometrial	HB	Blood	PCR-RFLP	91	261	0.681
Romanowicz-Makowska	2013	Poland	Caucasian	Endometrial	HB	Cervical specimens	PCR-RFLP	150	150	0.992
Cincin	2012	Turkey	Caucasian	Endometrial	HB	Blood	PCR-RFLP	104	158	0.396
Samulak	2011	Poland	Caucasian	Endometrial	HB	Cervical specimens	PCR-RFLP	456	300	0.505
Malisic	2015	Serbia	Caucasian	Ovarian	PB	Cervical specimens	PCR-RFLP	50	78	0
Monteiro	2014	Brazil	Mixed	Ovarian	HB	Blood	PCR-RFLP	70	70	0.676
Khokhrina	2015	Russia	Caucasian	Ovarian	HB	Blood	PCR-RFLP	104	298	0.908
Fan	2013	China	Asian	Cervical	HB	Blood	MAMA-PCR	235	350	0
Wang	2009	USA	Latino	Cervical	PB	Blood	Taqman	457	442	0.761
Wu	2003	Taiwan	Asian	Cervical	PB	Blood	PCR-RFLP	100	196	0.531
Settheetham-Ishida	2011	Thailand	Asian	Cervical	HB	Blood	PCR-RFLP	111	118	0.539
Huang	2007	China	Asian	Cervical	HB	Blood	MA-PCR	539	800	0.104
Farkasova	2008	Slovakia	Caucasian	Cervical	HB	Blood	PCR-RFLP	18	30	0.179
Djansugurova	2013	Kazakhstan	Mixed	Cervical	HB	Blood, cervical specimens	PCR-RFLP	217	160	0
Zhang	2012	China	Asian	Cervical	HB	Blood	SNPstream	80	177	0.538
Barbisan	2011	Argentine	Latino	Cervical	HB	Cervical specimens	PCR-RFLP	103	114	0.49
Jiang	2009	China	Asian	Cervical	HB	Blood	PCR-RFLP	436	503	0.482
Niwa	2005	Japan	Asian	Cervical	HB	Buffy coat	PCR-RFLP	131	320	0.088
Xiao	2010	China	Asian	Cervical	HB	Blood	PCR-RFLP	162	183	0.116
Roszak	2011	Poland	Caucasian	Cervical	PB	Blood	PCR-RFLP	189	308	0.371
Ma	2011	China	Asian	Cervical	HB	Blood	PCR-RFLP	200	200	0.061
Alsbeih	2013	SaudiArabia	Asian	Cervical	HB	Blood	Sequencing	100	100	0.04
Bajpai	2016	India	Indian	Cervical	PB	Blood, cervical specimens	PCR-RFLP	68	65	0.036
***rs1799782(Arg194Trp)***										
Michalska	2015	Poland	Caucasian	Ovarian	HB	Cervical specimens	PCR-RFLP	720	720	0.053
Monteiro	2014	Brazil	Mixed	Ovarian	HB	Blood	PCR-RFLP	70	70	0.69
Khokhrina	2012	Russia	Caucasian	Ovarian	HB	Blood	PCR-RFLP	104	298	0.562
Sobczuk	2012	Poland	Caucasian	Endometrial	HB	Blood	PCR-RFLP	94	114	0.588
Hosono	2013	Japan	Asian	Endometrial	HB	Blood	PCR-RFLP	91	251	0.525
Fan	2013	China	Asian	Cervical	HB	Blood	MAMA-PCR	235	350	0
Wu	2003	Taiwan	Asian	Cervical	PB	Blood	PCR-RFLP	100	196	0.196
Settheetham-Ishida	2011	Thailand	Asian	Cervical	HB	Blood	PCR-RFLP	111	118	0.023
Huang	2007	China	Asian	Cervical	HB	Blood	MA-PCR	539	800	0.731
Farkasova	2008	Slovakia	Caucasian	Cervical	HB	Blood	PCR-RFLP	17	30	0.543
Djansugurova	2013	Kazakhstan	Mixed	Cervical	HB	Blood, cervical specimens	PCR-RFLP	217	160	0.001
Zhang	2012	China	Asian	Cervical	HB	Blood	SNPstream	80	117	0.434
Barbisan	2011	Argentine	Latino	Cervical	HB	Cervical specimens	PCR-RFLP	103	114	0
Wang	2010	China	Asian	Cervical	HB	Blood	PCR-RFLP	123	175	0.849
Bajpai	2016	India	Indian	Cervical	PB	Blood, cervical specimens	PCR-RFLP	68	65	0.001
***rs25489 (Arg280His)***										
Wu	2003	Taiwan	Asian	Cervical	PB	Blood	PCR-RFLP	100	196	0.071
Huang	2007	China	Asian	Cervical	HB	Blood	MA-PCR	539	800	0.463
Zhang	2012	China	Asian	Cervical	HB	Blood	SNPstream	80	177	0.494
Wang	2010	China	Asian	Cervical	HB	Blood	PCR-RFLP	123	175	0.043
Bajpai	2016	India	Indian	Cervical	PB	Blood, cervical specimens	PCR-RFLP	65	68	0

Sourcecon: Source of control. HWEcon: Hardy–Weinberg equilibrium in controls. PB, population-based; HB, hospital-based.

Of the 24 studies related to rs25487, 16 focused on cervical cancer [14–29], 5 on endometrial cancer [30–34], and 3 on ovarian cancer [35–37]. The ethnic group was Asian in 11 studies and Non-Asian in the others. Of the 15 studies related to rs1799782, 10 focused on cervical cancer [16–20, 25, 27, 28], 3 on endometrial cancer [35, 37, 38], and 2 on ovarian cancer [30, 31]. The ethnic group was Asian in 7 studies and Non-Asian in 8 studies. Of the 5 studies related to rs25489, all focused on cervical cancer. The population was Asian in 4 studies and Non-Asian in one study.

Across all studies, the distribution of genotypes in controls was mostly in agreement with Hardy-Weinberg equilibrium (HWE), except in 5 studies on rs25487 [6, 15, 17, 18, 36], 5 studies on rs1799782 [15–18, 26] and 2 studies on rs25489 [15, 39].

### Quantitative data synthesis

Across the entire pooled study population, a significant association was not found between rs25487 and risk of female reproductive system cancer (Table 2). In the subgroup of Asian participants, however, we detected a significant association of the A variant at rs25487 with increased risk of female reproductive system cancer (GA vs. GG, OR 1.16, 95%CI 1.00–1.36). This association disappeared when we excluded 5 studies that deviated from HWE, instead appearing in the subgroup of Non-Asian participants (AA vs. GA/GG, OR 1.61, 95%CI 1.41–1.85). Subgroup analysis by cancer type indicated an association between the A allele at rs25487 and increased risk of cervical cancer (AA vs. GA/GG, OR 1.22, 95%CI 1.05–1.41), endometrial cancer (AA vs. GG, OR 2.16, 95%CI 1.00–4.67) and ovarian cancer (AA vs. GA/GG, OR 2.01, 95%CI 1.70–2.38). The significant associations with cervical cancer and ovarian cancer disappeared after removing studies that deviated from HWE.

**Table 2:** Meta-analysis of the associations between XRCC1 polymorphisms and risk of female reproductive system cancer

Variable	*N*	Cases/controls	Homozygous genetic model			Heterozygous genetic model			Dominant genetic model			Recessive genetic model		
			OR (95 % CI)	*Phet*	*I^2^*	OR (95 % CI)	*Phet*	*I^2^*	OR (95 % CI)	*Phet*	*I^2^*	OR (95 % CI)	*Phet*	*I^2^*
***rs25487 (Arg399Gln)***			AA vs. GG			GA vs. GG			(AA+GA) vs. GG			AA vs. (GA+GG)		
All studies	24	4265/5495	1.34(0.92,1.97)	0.000	81.6	1.06(0.92,1.22)	0.001	54.4	1.11(0.95,1.31)	0.000	67.2	1.32(0.89,1.95)	0.000	85.1
Ethnicity														
Asian	11	2185/3208	1.49(0.90,2.45)	0.000	73.6	1.16(1.00,1.36)	0.121	34.7	1.19(0.99,1.42)	0.014	55.1	1.41(0.87,2.27)	0.000	72.8
Non-Asian	13	2080/2287	1.24(0.68,2.24)	0.000	86.5	0.96(0.76,1.22)	0.004	59.7	1.03(0.79,1.36)	0.000	73.5	1.26(0.68,2.32)	0.000	89.9
Tumor type														
Cervical	16	3146/4066	1.36(0.87,2.12)	0.000	80.5	1.10(0.93,1.30)	0.000	58.5	1.11(0.92,1.33)	0.000	68.6	1.22(1.05,1.41)	0.000	79.0
Endometrial	5	895/983	2.16(1.00,4.67)	0.006	72	1.02(0.67,1.53)	0.022	65.1	1.37(0.92,2.03)	0.016	67.0	0.68(0.43,1.07)	0.000	84.1
Ovarian	3	224/446	0.63(0.21,1.93)	0.026	72.5	0.92(0.65,1.31)	0.921	0.0	0.80(0.51,1.25)	0.186	40.6	2.01(1.70,2.38)	0.025	72.8
Consistent with HWE	19	3595/4742	1.35(0.94,1.92)	0.000	75.6	1.07(0.90,1.27)	0.000	63.7	1.16(0.97,1.38)	0.000	68.5	1.31(0.90,1.91)	0.000	81.6
Asian	9	1850/2758	1.17(0.76,1.80)	0.009	60.7	1.18(0.99,1.42)	0.073	44.2	1.16(0.94,1.44)	0.005	63.9	1.10(0.76,1.62)	0.036	51.5
Non-Asian	10	1745/1984	1.59(0.90,2.80)	0.000	83.1	0.97(0.72,1.30)	0.001	69.7	1.15(0.85,1.56)	0.000	73.7	1.61(1.41,1.85)	0.000	87.9
Tumor type (consistent with HWE)														
Cervical	12	2526/3391	1.17(0.80,1.71)	0.000	69.6	1.11(0.90,1.38)	0.000	69.3	1.11(0.89,1.39)	0.000	73.5	1.10(0.93,1.30)	0.010	56.7
Endometrial	5	895/983	2.16(1.00,4.67)	0.006	72.0	1.02(0.67,1.53)	0.022	65.1	1.37(0.92,2.03)	0.016	67.0	0.68(0.43,1.07)	0.000	84.1
Ovarian	2	560/368	1.04(0.55,1.99)	0.582	0.0	0.95(0.64,1.41)	0.950	0.0	0.97(0.67,1.40)	0.907	0.0	1.06(0.61,1.84)	0.557	0.0
***rs1799782 (Arg194Trp)***			TT vs. CC			CT vs. CC			(TT+CT) vs. CC			TT vs. (CT+CC)		
All studies	15	2672/3578	1.19(0.67,2.13)	0.000	84.7	1.02(0.84,1.23)	0.011	52.5	0.93(0.71,1.21)	0.000	77.1	1.19(0.71,1.98)	0.000	82.5
Ethnicity														
Asian	7	1279/2007	2.30(1.39,3.82)	0.016	61.4	1.16(0.99,1.34)	0.588	0.0	1.28(1.10,1.50)	0.348	10.7	2.11(1.33,3.34)	0.031	56.9
Non-Asian	8	1393/1571	0.42(0.14,1.26)	0.000	83.5	0.76(0.48,1.19)	0.003	70.4	0.62(0.39,0.99)	0.000	75.7	0.46(0.20,1.10)	0.001	76.1
Tumor type														
Cervical	10	1593/2125	1.20(0.50,2.87)	0.000	88.7	1.02(0.79,1.32)	0.008	61.4	0.96(0.68,1.36)	0.000	80.7	1.30(1.07,1.59)	0.000	86.6
Endometrial	3	185/365	2.50(1.16,5.37)	/	/	1.01(0.36,2.87)	0.079	67.5	1.06(0.34,3.31)	0.054	73.1	1.80(0.98,3.29)	/	/
Ovarian	2	894/1088	0.96(0.72,1.28)	0.789	0.0	0.93(0.62,1.38)	0.222	33.6	0.77(0.62,0.95)	0.755	0.0	0.91(0.77,1.09)	0.831	0.0
Consistent with HWE	10	1938/2771	1.45(0.96,2.19)	0.017	58.9	1.08(0.91,1.28)	0.276	18.8	1.01(0.79,1.29)	0.009	58.7	1.35(0.90,2.02)	0.009	62.4
Asian	5	933/1539	1.67(1.33,2.09)	0.224	29.6	1.08(0.98,1.18)	0.311	16.3	1.12(1.04,1.21)	0.153	40.2	1.65(1.30,2.09)	0.403	0.6
Non-Asian	5	1005/1232	0.98(0.85,1.13)	0.782	0.0	1.00(0.92,1.10)	0.227	30.9	0.93(0.88,0.98)	0.504	0.0	0.91(0.77,1.09)	0.823	0.0
Tumor type (consistent with HWE)														
Cervical	5	859/1318	1.62(1.26,2.07)	0.156	42.5	1.06(0.95,1.17)	0.337	11.1	1.10(1.01,1.20)	0.243	26.8	1.63(1.26,2.11)	0.268	23.9
Endometrial	3	185/365	2.00(1.15,3.49)	/	/	1.12(0.89,1.41)	0.120	58.6	1.15(0.96,1.39)	0.110	60.9	1.80(0.98,3.29)	/	/
Ovarian	2	894/1088	0.98(0.85,1.13)	0.782	0.0	1.01(0.93,1.11)	0.257	26.5	0.93(0.88,0.99)	0.355	3.5	0.91(0.77,1.09)	0.823	0.0
***rs25489 (Arg280His)***			AA vs. GG			GA vs. GG			(AA+GA) vs. GG			AA vs. (GA+GG)		
All studies	5	907/1416	2.91(1.17,7.26)	0.067	54.3	0.98(0.80,1.21)	0.558	0.0	1.31(0.77,2.24)	0.000	80.5	3.16(1.91,5.24)	0.093	49.7
Ethnicity														
Asian	4	842/1348	1.73(0.87,3.43)	0.524	0.0	0.97(0.82,1.15)	0.683	0.0	1.00(0.85,1.18)	0.546	0.0	1.74(0.88,3.45)	0.512	0.0
Non-Asian	1	65/68	3.10(1.85,5.20)	/	/	1.81(0.68,4.86)	/	/	2.35(1.57,3.52)	/	/	3.14(1.85,5.32)	/	/

N: Number of studies. P_het_: *P* value for heterogeneity test. HWE: Hardy–Weinberg equilibrium.

Across the entire pooled study population, no association was found between rs1799782 and risk of female reproductive system cancer (Table 2). In subgroup analysis by ethnicity, the T variant was significantly associated with increased risk of female reproductive system cancer in Asians (TT vs. CC, OR 2.30, 95%CI 1.39–3.82; TT/CT vs. CC, OR 1.28, 95%CI 1.10–1.50; TT vs. CT/CC, OR 2.11, 95%CI 1.33–3.34). This association remained significant after excluding 5 studies that deviated from HWE(TT vs. CC, OR 1.67, 95%CI 1.33–2.09; TT/CT vs. CC, OR 1.12, 95%CI 1.04–1.21; TT vs. CT/CC, OR 1.65, 95%CI 1.30–2.09). In subgroup analysis by tumor type, the T allele was associated with increased risk of cervical cancer (TT vs. CT/CC, OR 1.30, 95%CI 1.07–1.59), and this association remained significant after excluding studies that deviated from HWE (TT vs. CC, OR 1.62, 95%CI 1.26–2.07; TT/CT vs. CC, OR 1.10, 95%CI 1.01–1.20; TT vs. CT/CC, OR 1.63, 95%CI 1.26–2.11). The same T variant increased risk of endometrial cancer based on all study participants (TT vs. CC, OR 2.50, 95%CI 1.16–5.37) as well as based on only studies consistent with HWE (TT vs. CC, OR 2.00, 95%CI 1.15–3.49).

Data on rs25489 SNPs were limited to cervical cancer studies. Meta-analysis suggested that the A variant was associated with increased risk of this cancer (AA vs. GG, OR 2.91, 95%CI 1.17–7.26; AA vs. GA/GG, OR 3.16, 95%CI 1.91–5.24).

### Heterogeneity and sensitivity analyses

Significant heterogeneity across studies was observed in the meta-analysis of the association between the A variant at rs25487 and risk of female reproductive system cancer (homozygous model, I^2^ = 81.6, *P* = 0.000; heterozygous model: I^2^ = 54.4, *P* = 0.001; dominant model: I^2^ = 67.2, *P* < 0.001; recessive model: I^2^ = 85.1, *P* < 0.001). Similarly, significant heterogeneity across studies was observed in the meta-analysis of the association between the T allele at rs1799782 and cancer risk (homozygous model, I^2^ = 84.7, *P* < 0.001; heterozygous model, I^2^ = 52.5, *P* = 0.011; dominant model, I^2^ = 77.1, *P* < 0.001; recessive model: I^2^ = 82.5, *P* < 0.001). Among studies used in meta-analyses involving rs25489, we found moderate heterogeneity in the homozygous model (I^2^ = 54.3, *P* = 0.067) and dominant model (I^2^ = 80.5, *P* < 0.001), but no significant heterogeneity in the heterozygous or recessive models.

Then we performed sensitivity analysis, in which we recalculated the meta-analysis after deleting each study systematically. The results were not substantially different after excluding any single study, indicating the robustness of our original meta-analyses.

## DISCUSSION

XRCC1 is the first protein to participate in the BER pathway, acting as a scaffold for other DNA repair proteins, such as DNA ligase IIIa, DNA polymerase β and poly (ADP-ribose) polymerase [40]. The *XRCC1* SNPs rs25487, rs1799782, and rs25489 have been linked to susceptibility for several types of cancer, but it is unclear whether this is also true for female reproductive system cancer. The present meta-analysis suggests that as in other cancers, *XRCC1* polymorphism may also influence tumorigenesis in the female reproductive system.

The present study may be the first quantitative meta-analysis of *XRCC1* polymorphism and risk of female reproductive system cancer. Previous meta-analyses focused only on cervical cancer risk [41–45], and they reported that rs25487 and rs1799782 were associated with increased risk in Asian populations. The present meta-analysis extended this finding by showing that in Asians, the A allele of rs25487 and T allele of rs1799782 are associated with increased risk of female reproductive system cancer. These findings should be verified in larger studies, especially since the association with rs25487 disappeared in Asians and appeared in Non-Asians after we excluded studies deviating from HWE.

We did not observe a significant association between *XRCC1* rs25489 and risk of ovarian cancer, even though estrogens and their metabolites damage DNA by forming bulky DNA adducts [46], which are normally repaired by the BER pathway. It is possible that our negative results reflect limited sample size. Further studies are needed to verify our findings.

To ensure results as reliable as possible, we analogized the studies in our meta-analysis to approximate randomized controlled trials. At the same time, our study does have several limitations. Our results were based on OR analyses that did not adjust for age, family history, gender, reproductive history, or other biological factors that might influence risk of female reproductive system cancer. Similarly, we did not take into account potential effects of gene-environment interaction. Therefore further work is needed before definitive conclusions can be drawn about these three *XRCC1* SNPs and risk of female reproductive system cancer.

Despite these limitations, our data provide up-to-date evidence from a comprehensive review of the literature that the A allele of rs25487 and T allele of rs1799782 are low-penetration risk factors for female reproductive system cancer in Asians. It may be that these alleles translate to weaker interaction between XRCC1 and other repair proteins, thereby reducing DNA repair capacity [47]. Our findings add to the growing evidence that polymorphism in DNA repair genes can destabilize the genome and increase tumor susceptibility [32].

## MATERIALS AND METHODS

### Literature search strategy

We performed a comprehensive literature search in the PubMed, EMBASE, Scopus, and CNKI databases up to December 17, 2016. The following search strings were used: “X-ray repair cross complementing protein1” or “XRCC1”; “polymorphisms” or “variants”; “carcinoma” or “cancer” or “malignancy” or “neoplasm” or “tumour” or “tumor”; “cervical” or “endometrial” or “ovarian” or “vaginal” or “vulvar” or “fallopian tube” or “female reproductive system”. The reference lists of relevant articles were also searched manually to identify additional eligible studies. When different studies presented overlapping data, we included only the larger study.

### Inclusion and exclusion criteria

Studies included in this meta-analysis had to be case-control or genome-wide association studies for which full text was available and that reported adequate data on genotype frequencies for cases and controls. Studies were excluded if they reported data overlapping with those of a larger study.

### Data extraction

Two investigators (N.-N.Y and Y.-F.H) independently extracted the following data from all eligible publications: the first author's name, year of publication, country of origin, ethnicity, study type (retrospective or prospective), source of control subjects (population-based [PB] or hospital-based [HB]), DNA source (*e.g*., blood, lymphocytes or buffy coat), genotyping method, total numbers of cases and controls and *P value* for HWE. Conflicts were resolved by consensus among all authors.

### Statistical analysis

Agreement between study results and HWE predictions was tested using the goodness-of-fit χ^2^ test, with the threshold for HWE defined as *P* > 0.05. Strength of association between *XRCC1* SNPs and cancer risk was assessed using odds ratios (ORs) and associated 95% confidence intervals (95%CI). Four different genetic models were conducted to detect the association: homozygous model (VV vs. WW), heterozygous model (WV vs. WW), dominant genetic model (VV+WV vs. WW) and recessive model (VV vs. WW+WV), with W and V representing the wild and variant alleles of each SNP. Heterogeneity across studies was assessed using a χ^2^-test-based *Q* statistic test, and the level of heterogeneity was quantified using the I^2^ test. When heterogeneity across studies was obvious (*P* ≥ 0.05 or I^2^ < 50%) [48], the random effects model was used to meta-analyze data from different studies. Otherwise, the fixed effects model was adopted [49]. All studies were analogized into interim randomized controlled clinical trials in order to control for type I and type II error.

Subgroup analyses were carried out based on ethnicity, tumor type and HWE [50]. Sensitivity analysis was performed in which we recalculated the meta-analysis after deleting each study systematically. Publication bias was investigated using Begg's and Egger's test [51], with significant risk of bias defined as *P* < 0.05. All statistical analyses were performed using STATA 11.0 (Stata Corp, College Station, Texas USA). All *P* values were two-sided.
